# COVID-19 - ESSKA guidelines and recommendations for resuming elective surgery

**DOI:** 10.1186/s40634-020-00248-4

**Published:** 2020-05-13

**Authors:** Caroline Mouton, Michael T. Hirschmann, Matthieu Ollivier, Romain Seil, Jacques Menetrey

**Affiliations:** 1 Sports Clinic, Centre Hospitalier de Luxembourg – Clinique d’Eich, 78, rue d’ Eich, 1460 Luxembourg, Luxembourg; 2 Luxembourg Institute of Research in Orthopaedics, Sports Medicine and Science, Luxembourg, Luxembourg; 3 Department of Orthopaedic Surgery and Traumatology, Kantonsspital Baselland (Bruderholz, Liestal, Laufen), CH-4101 Bruderholz, Switzerland; 4 Department of Orthopedics and Traumatology, Aix Marseille Univ, APHM, CNRS, ISM, Sainte-Marguerite Hospital, Institute for Locomotion, Marseille, France; 5 Centre de Médecine du Sport et de l’Exercice (CMSE), Swiss Olympic Medical Center, Hirslanden Clinique La Colline, Chemin Thury 7A, CH-1206 Geneva, Switzerland; 6 Division of Orthopaedic Surgery, University Hospital of Geneva, Geneva, Switzerland

## Abstract

The roadmap to elective surgery resumption after this COVID-19 pandemic should be progressive and cautious. The aim of this paper was to give recommendations and guidelines for resuming elective orthopedic surgery in the safest environment possible. Elective surgery should be performed in COVID-free facilities and hospital stay should be as short as possible. For matters of safety, patients considered first for surgery should be carefully selected according to COVID infection status/exposure, age, ASA physical status classification system / risk factors, socio-professional situation and surgical indication. A strategy for resuming elective surgery in four phases is proposed. Preoperative testing for COVID-19 infection is highly recommended. In any cases, COVID symptoms including fever and increased temperature should be constantly monitored until the day of surgery. Elective surgery should be postponed at the slightest suspicion of a COVID-19 infection. In case of surgery, adapted personal protective equipment in terms of gowns, gloves, masks and eye protection is highly recommended and described.

## Introduction

Since March 2020, hospitals and surgeons have been advised to postpone or cancel elective scheduled operations until the inflection point in the exposure graph has passed so that healthcare infrastructures can support the critical patient care needs [1]. Hospital resources have therefore mainly been dedicated to the care of COVID-19 patients resulting in thousands of orthopedic surgeries being delayed all over the world if they were not considered to cause a significant harm to the patient or outcome.

It is commonly agreed that there should be a decrease in incidence of COVID-19 cases for at least 2 weeks before elective surgery is resumed [2]. In Europe, several countries will soon reach this goal. Although the sanitary crisis can be considered to be under control, one should keep in mind that the pandemic is not finished yet. Premature and sudden lifting of interventions could lead to a second wave of infections [3]. It is thus critical to maintain regulatory measures within healthcare infrastructures within the next months to assure patient and staff safety and limit the number of infections. This can only be achieved by preparing the roadmap to elective surgery resumption. Unfortunately, there is, to date, no specific literature concerning the organization of an outpatient clinic and surgical activities [4].

The aim of this paper was therefore to provide recommendations and guidelines for resuming elective orthopedic surgery in this period of pandemic in the safest environment possible and according to the current state of knowledge on COVID-19.

## Surgeries covered by the present guideline

The American Academy of Orthopaedic surgeons defined four type of orthopaedics procedures in times of pandemic: A) emergency only, B) urgent types of surgeries, C) urgent/somewhat elective and D) elective (Table 1). The current guideline only covers categories A and B of this definition. These include all types of surgeries with predominantly chronic conditions or acute cases where surgery generally can be delayed without causing serious harm to patients. More precisely, the following procedures are considered: tendinous or ligamentous intra- or periarticular injuries of chronic and acute in nature; deformity-correcting osteotomies; joint arthroplasty procedures; elective shoulder, elbow, hand, hip, knee and foot and ankle surgery.

**Table 1:** Adapted from AAOS guidelines for elective surgery [5]

Category	A	B	C	D
Degree of emergency	Emergency	Urgent	Urgent/ Somewhat elective	Elective
Types of procedures (non-exhaustive selection)	Life- or limb-threatening conditions	Joint/arthroplasty infections Most trauma cases	Acute intra- and periarticular ligament & tendon conditions (e.g. ACL tears, meniscus bucket handle tears) Selected trauma cases	Total joint arthroplasty Osteotomies Chronic intra- and periarticular ligament & tendon conditions Chronic peripheral nerve compression syndromes

The present guideline is essentially based upon a review of sparse literature, interviews of specialist and mostly expert opinion. The content has been reviewed by a scientific committee. It can be useful in conducting negotiations or for practical implementation at the level of a hospital or a national specialty society but cannot be imposed as official guidelines. The purpose of this guideline is only to provide the community with possible answers as well as to raise awareness of the complexity of the problem.

## Before resuming surgical activities

The main objective before resuming any surgical activity is to assure that all necessary regulatory measures are in place within the healthcare infrastructure to allow for the safety of patients, physicians, staff and institution until the end of the pandemic. All decision in the organizational aspect should naturally be locally based decisions, follow legal restrictions and guidelines from the relevant health authorities.

First, state and hospital regulations should allow for a return to outpatient clinic and elective surgeries. These regulations/rules should be carefully reviewed to identify any aspect that may impact the ability of the facility to provide services. The facility’s liability and malpractice insurance should also be confirmed for the emergent services. Ideally, a permanent COVID-negative clinical pathway should be devoted to elective surgery or urgent, somewhat elective surgery. This could be an isolated institution, a separate COVID free building of the institution or made possible with the creation of additional space. Sufficient space should also be available to allow for social distancing protocols.

All collaborators should be tested (COVID-kit test, immune/serology test if possible) before resumption of clinical practice. According to the development of the pandemic, they should be monitored on a regular (e.g. weekly) basis and their protection be guaranteed (supplies, PPE). Testing of patients should also be organized before any surgery to avoid importing asymptomatic cases within the COVID-negative clinical pathway as well as to prevent secondary surge. Prescreening of exposed patients is of importance to avoid the planning of surgeries during the incubation period of COVID-19 and adequate policy should be implemented to avoid peri-operative COVID infection. Finally, care should be taken to operate patients when a standardized and sufficient postoperative follow-up is assured. Nurses, the team of anesthesiology, physiotherapists should be consulted and aware that elective surgeries will start again to guarantee proper patient care.

## Preselection of patients eligible for surgery

When resuming surgical activities, a careful selection of patients should be implemented in order to limit the risk of infections and complications. The selection of prioritized patients should take into account several parameters such as COVID exposure, age, American Society of Anesthesiologists (ASA) physical status classification system / risk factors and socio-professional situation.

According to Fineberg [6], patients should be treated according to their COVID-19 exposure. The author defined five types of patient categories: A patient (1) who is not known to have been exposed or infected, (2) who has been exposed but is asymptomatic, (3) who has recovered from infection and maybe adequately immune, (4) who is possibly infected (persons with sign and symptoms consistent with infection who initially test negative), (5) who is infected.

Age and comorbidities are recognized to be negatively associated with outcomes of COVID-19. They should be taken into account in evaluating the risk to operate a patient. In the analysis of 44,672 confirmed cases in China, 81% of all death after COVID-19 infection occurred in patients above 60 years old [7]. A meta-analysis including 30 studies and 53,000 patients revealed that age (above 50), smoking, and any comorbidity were significant predictors for disease severity [8]. Other comorbidities to consider include high blood pressure, cardiovascular diseases, diabetes, lung diseases, cancer, liver and kidney diseases [9]. Patients with BMI above [10] 30 kg/m2 may also be considered at risk for severe forms of COVID-19. Finally, the socio-professional situation should be considered with probably a priority for active workers. Other aspects such as the presence at home from a person at risk for COVID-19 may also be taken into account.

To summarize, Table 2 presents the stratification of the theoretical risk of infection with respect to patient age, category according to Fineberg [6] and comorbidities. A sixth category of patients was added to take into account the presence of comorbidities. To resume elective surgery, patients with no sign of COVID-19 or who have recovered from the disease (categories 1, 2, 3) should have the priority. Patients with COVID-19 infection (suspected or confirmed: category 4, 5, 6) should have fully recovered and displayed an adequate immune response before considering surgery. ASA I (normal healthy patient) and II (patient with a mild systemic disease) should also have the priority. Any risk factor or comorbidity (e.g. age > 60 years, obesity, high blood pressure, cardiovascular disease and diabetes) should be disqualifying conditions in the early phase of elective surgery resumption.

**Table 2:** Stratification of the theoretical risk of infection with respect to patient age, category according to Fineberg [11] and comorbidities

Category/Age	< 40	40–60	60–70	70+
1				+
2			+	++
3		+	++	+++
4	+	++	+++	++++
5	+	++	+++	++++
6	++	+++	++++	+++++

## Implication of surgical indications in patient selection

In the early phase, patient selection should also include the indication for surgery. The viral SARS Cov-2 concentration in articular, periarticular and bony tissues and fluids of infected patients is currently unknown. However, it is reasonable to assume that it is lower in musculoskeletal tissues than in respiratory or digestive tissues. Given these uncertainties, it is recommended to decrease those types of surgeries which generate a high amount of aerosol like electrocautery, working with oscillating saws as well as pulse lavage procedures [12]. In the absence of well-established scientific criteria, minimally invasive and arthroscopic may have the lowest infection risk and currently appear to be safer. They should be considered a priority if elective surgeries will be resumed. Surgical procedures followed by a hospital stay of no more than two to three days should be initially favored. Surgeries with a higher degree of invasiveness and blood loss may follow later.

## Overview of patient selection process

Currently, it appears that patients of categories 1–3 (combining the criteria young age, absence of comorbidities, Table 2) and minimally invasive or arthroscopic surgery may have the lowest risk both for patients and surgeons. A strategy for resuming elective surgery may thus be foreseen in 4 phases: (1) Mini-invasive surgeries for patients under 60 years old, with no comorbidity and an hospital stay of maximum 3 days, (2) Surgeries for all patients without comorbidities and an hospital stay of maximum 3 days, (3) Mini-invasive surgeries for patients under 60 years old, with comorbidities or with an hospital stay above 3 days, (4) Surgeries for all patient with comorbidities (Table 3). Within each of these phases, two scenarios should be considered through a careful preoperative screening of patients. Appropriate scheduling should be made according to the COVID status and patients with no sign of COVID-19 or who have recovered from the disease (categories 1, 2, 3) should be separated from patients with COVID-19 infection (suspected or confirmed: category 4, 5, 6). The surgery should be delayed by a minimum of 8 weeks for the latter (until full recovery).

**Table 3:** Strategy for resuming elective surgery in 4 phases according to patient age, comorbidities, type of surgery and length of hospital stay

Priority	1		2		3		4	
Scenario	A	B	C	D	E	F	G	H
Patient age	< 60	< 60	All ages	All ages	< 60	< 60	All ages	All ages
Surgery	Mini-invasive	Mini-invasive	Mini invasive and open surgery	Mini invasive and open surgery	Mini-invasive	Mini-invasive	Mini invasive and open surgery	Mini invasive and open surgery
Length of stay	Maximum 3 days	Maximum 3 days	Maximum 3 days	Maximum 3 days	All	All	All	All
Comorbidities	None	None	None	None	None / Existing	None / Existing	Existing	Existing
Covid-19	No risk detected / Potentially recovered	Infected / With symptoms	No risk detected / Potentially recovered	Infected / With symptoms	No risk detected / Potentially recovered	Infected / With symptoms	No risk detected / Potentially recovered	Infected / With symptoms

## Pre-operative screening

Patient should be screened preoperatively (e.g. by tele-or videoconference). A detailed discussion with the patient about his/her situation may be organized and includes COVID-19 related questions (symptoms, exposure, previous testing). Patients must understand all aspects of his/her medical care that may change due to the current health emergency situation and also be aware about the limited but existent risks of COVID-19 infection during surgery / hospitalization. Despite all the preventive measures organized by the facilities to avoid COVID-19 infections during surgery and/or hospitalization, the infection risk can indeed not be 100% excluded. The patient should thus agree to undergo surgery under these conditions as well as additional preoperative screening. If a suspicion or confirmation of COVID-19 exists after the preoperative screening, the surgery should be delayed.

In any cases, COVID symptoms including temperature should be constantly monitored until the day of surgery. Patients who are not known to have been exposed or infected (category 1) should receive at least receive a COVID-19-RT-PCR test 48 to 72 h before the operation. Patients who have been exposed but asymptomatic (category 2) should get a COVID-RT-PCR test, and eventually lung CT-scan 48 to 72 h before surgery. Patients who have recovered from infection and maybe adequately immune (category 3) should get a validated immune/serology test if allowed and available 48 to 72 h before surgery. Patients presumed to be infected (category 4) should get a repeated COVID-RT-PCR test, and eventually lung CT-scan before considering any surgeries. Finally, surgery of infected patients (categories 5 and 6) should be delayed until full recovery (at least 2 months) and should get COVID-RT-PCR test, and eventually lung CT-scan 48 to 72 h before surgery.

A systematic review reported that 2588 out of 2866 (88.2%) patients displayed abnormalities on lung CT-scan which places this technique as a good candidate to detect COVID-19 patients [11]. Known features of COVID-19 on lung CT-scan mainly include bilateral (87.5%, 435 out of 497 patients), multilobular (78.8%, 108 out of 137), ground-glass opacification (GGO; 88%, 346 out of 393) with a peripheral (76.0%, 92 out of 121) or posterior distribution (80,4%, 41 out of 51) [13]. It has been shown that some patients with a negative COVID-RT-PCR test displayed abnormalities on lung CT-scan [14], questioning the sensitivity of the RT-PCR test. It appears that the combination of RT-PCR and lung CT-scan lead to the highest diagnostic sensitivity [15] (92%) compared to RT-PCR alone (78%), lung CT-scan alone (67%) or combination of 2 RT-PCR (86%). Combination of both RT-PCR test and lung CT-scan to exclude COVID-19 patients is thus recommended to limit the risk to bring infected patient to surgery.

Other tests, depending on facility rules, may include stool culture as a meta-analyses reported that viral RNA could be found in 48,1% of stool samples (95% CI, 38.3%–57.9%) [16]. An interesting fact is that 70.3% of the positive samples were collected after loss of virus detection from respiratory specimens. Stool culture may therefore be of interest after COVID-19 infection to insure that the patient is free from virus.

Before surgery, blood samples should be analyzed to detect any abnormalities such as an increased in C-reactive protein and modified blood count. A meta-analysis of 19 publications [17] showed that common laboratory findings in COVID-19 patients (observed in more than half of studied patients) included: decreased albumin (75,8%; 95% CI: 30,5–100,0), high C-reactive protein (58,3%; 95% CI: 21,8–94,7), high lactate dehydrogenase (57,0%; 95% CI: 38,0–76,0), lymphopenia (43,1%; 95% CI: 18,9–67,3).

## Anesthesia

Prevention of the coronavirus through aerosolization in a potentially infected patient should be the priority [18]. Therefore, it appears that local/regional anesthesia should be preferred to invasive airway management whenever possible for elective orthopaedic procedures of the upper and lower extremity. Spinal anesthesia is reported to be safe, even in COVID positive patients [19]. If possible, patients should wear surgical masks during the procedure. In case of anesthetic procedures with invasive airway management, surgeons should be aware that aerosolization through exhaled gases may occur. After the procedure, coughing on emergence should be minimized or avoided, as well as postoperative nausea and vomiting. Routine thrombo-prophylaxy should be meticulously prescribed for the time being between the operation and the authorization for partial to full weight-bearing.

## Recommended personal protective equipment (PPE)

Balanced recommendations for PPE in operating area for COVID-19 positive patients or suspected COVID-19 patients are found in Table 4 [12]. Sterile surgical gowns are part of the standard protection in the OR. In every surgery the OR team consisting of the surgeon, the surgical assistants and the scrub nurse wear sterile surgical gowns in order to reduce intraoperative wound contamination and to minimize the patient’s infection risk. It is also a personal protection against blood and body fluids which often spray in an area of 3–8 m around the operating table. The safety levels of gowns for medical use can be classified in levels 1–4 [20]. Level 1 gowns should be used in minimal risk environment such as basic care or for visitors. Level 2 gowns should be used in low risk procedures such as venous blood draw. Level 3 gowns are generally used for moderate risk procedures such as arterial blood draw, or in the ER. Level 4 gowns are preserved for high risk procedures such as surgery or when infectious diseases are suspected.

**Table 4:** Balanced recommendations for PPE in operating area for COVID-19499 positive patients or suspected COVID-19 patients [12]

	Health care personal (HCP)	Masks		Surgical gowns	Eye protection	Gloves
		Surgical	FFP1–3 N95–100			
**Patient transport in and from OR**	Persons involved in transport of patients	X	–	Level 1	–	X
**Transfer of patient into OR area**	All HCP	X	–	Level 1	X	X
**Intubation and initiation of anesthesia in OR**	All HCP in OR	–	>FFP2/N95FFP3 N99	>Level 3	X when distance < 2 m	X
**Surgery including surgical AGPs**	All HCP in OR	–	>FFP2/N95FFP3 N99	>Level 3	X when distance < 2 m	X (double gloving)
	Occupational department personal (ODP)	X		>Level 3	X	X
**Surgery including respiratory AGPs**	All HCP in OR		>FFP2/N95FFP3 N99 or PAPR^a^ if surgeon needs it	>Level 3	X when distance < 2 m	X
	Occupational department personal (ODP)	X	–	>Level 3	X	X
**Extubation and ending of anaesthesia in OR**	All HCP in OR	–	>FFP2/N95FFP3 N99	>Level 3	X when distance < 2 m	X
**Cleaning of OR**	Cleaning personal	–	>FFP2/N95FFP3 N99	>Level 3	X	X

^a^Powered air-purifying respirator, X = indicated, − = not indicated

Helmets or togas might also be an option for protection against body spray, but only protect against airborne transmission of COVID-19 in combination with respirator masks. There are three different types of disposable masks available. Single-use face masks, surgical masks, and respiratory masks. Single use face masks, which are typically thin and consisting of only one layer are only capable of filtering rather larger particles (3 μm). Surgical masks are generally more effective than single use face masks in filtering virus-sized particles. A medical or surgical mask may be sufficient to prevent droplet transfer but do not offer protection against high risk AGPs, while a respirator mask is required for airborne infection. Air purifying respirator masks should thus be used as they are generally filtering smaller sized particles (0.3 μm) than surgical masks. The European Standard (EN 149:2001) classifies respirator masks into three different categories: Filtering facepiece 1 (FFP1), FFP2, and FFP3. FFP2 is comparable to US standard N95 [21]. Respiratory AGP require FFP3 masks or powered air-purifying respirators, whereas surgical AGP only require FFP-2 masks. The fitting and sizing of the mask is of utmost importance. Only a perfect sized and well fitted mask leads to efficient sealing of the respiratory tract.

Eye protection is critical for orthopaedic surgeons as many procedures such as the use of power tools frequently lead to contamination of every OR personal in the room and surface contamination in the OR in an area of up to six meters around the operating table.

## Post-operative follow-up

To assure a proper post-operative management of elective cases, the country, the region, the city should be partially or completely reopened with notably a reopening of physical therapy and outpatient facilities. Postoperative appointments should be planned in the early postoperative phase to detect potential COVID-related complications and made, if possible, with the use of videoconference and/or telehealthcare to minimize repetitive post-operative visits and therefore limit patient displacement.

If telehealthcare cannot be considered for some patients, the facility should organize a specific flow of patients to allow distancing protocol. This can include a minimization of patient number, maintaining a gap between booked appointments, preparing the waiting room with enough space for social distancing protocols and organize the patient itinerary to avoid patients to come across from each other.

## Conclusion

The roadmap to elective surgery resumption should be progressive and cautious, especially in area of pandemic focus. Elective surgery should ideally be strictly performed in a COVID-free facility and hospital stay should be as short as possible. For matter of safety, patients operated first should be carefully selected according to COVID exposure, age, ASA physical status classification system / risk factors, socio-professional situation and surgical indication. At the slightest suspicion of COVID symptoms, elective surgery should be postponed. In case of surgery, close monitoring of COVID-19 signs and adapted personal protective equipment is highly recommended.

## Supplementary information

**Additional file 1.** Facility checklist to resume elective surgery.

**Additional file 2.** What should I inform my patient about before undergoing surgery?

**Additional file 3.** Preoperative screening of patients.

**Additional file 4.** Which patients should you begin with for elective orthopaedic surgery?
