# Supplementary material for: COVID-19 - ESSKA guidelines and recommendations for resuming elective surgery
Source: J Exp Orthop. 2020 May 13;7:28. doi: 10.1186/s40634-020-00248-4 (PMC7220621; doi:10.1186/s40634-020-00248-4)
Supplement: Supplementary file 1 — Additional file 1. Facility checklist to resume elective surgery. [file 40634_2020_248_MOESM1_ESM.pdf]

Facility Checklist to Resume Elective Surgery

Chapters	Questions	Yes	No	Limitations or further comments
**COVID Pandemic situation**	Is your state regulation allowing a return to outpatient clinic and/or elective surgery?			
	Is there a need in your community to create additional space?			
	Is your local hospital/emergency Pandemic management committee informed that your clinic will restart elective surgery?			
	Have you confirmed there are no state emergency preparedness or pandemic rules that will prohibit the facility from providing outpatients or elective surgery services?			
	Have you reviewed your state rules/regulations that might impact the facility's ability to provide services under the blanket waivers?			
**Facility specificity**	Have you confirmed your facility's liability and malpractice insurance covers enhanced emergent services?			
	Have you considered what hours the facility would be available to accept your patients?			
	Has your anaesthesiology team been notified of the restart?			
	Has your nurse team been notified of the restart?			
	Is your facility a COVID-free structure?			
	If not, is your facility providing a safe workflow avoiding any contact with COVID patients?			
	Would your facility be able to accommodate meals for overnight patients?			
	Does your facility have enough space for social distancing protocols?			
	Can your facility provide 24-hour coverage for patients who need to stay?			
	Does your facility have the space to have patients stay overnight? If yes, how many patients could your facility accommodate?			
**Workers Protections**	Does your facility have the supplies needed for worker protection?			
	Does your facility have the PPE needed for COVID + situation?			
	Can your facility provide patients appropriate lab testing and results?			

Published 22.04.2020

**Patients Protection**	Can your facility provide workers appropriate lab testing and results?			
	Can your facility provide the appropriate pathology/comorbidities testing and results?			
	Does your facility have the supplies needed for patient’s protection?			
	Has your facility decided which worker is allowed to receive patients, work in outpatient clinic and/or OR, and peri-operative cares (immunized, iterative testings?)?			
**OR: elective surgery**	Does your facility have the appropriate number of anaesthesia machines to restart elective procedures?			
	Does your postoperative area have enough room to allow a minimal patient's distancing?			
	Does your facility have the ability or specific supply chain to sterilize necessary equipment?			
	Does the facility have the appropriate staffing to support these elective procedures? (clinical, sterile processing dept., administrative, etc.)			
	Has your facility decided upon which type of surgery and which group of patients might benefit (first) from elective surgery restart?			
	Does your facility have the ability or specific supply chain to monitor, prepare anaesthesia and potentially have access to an ICU Bed (if something bad happen)?			
**Outpatient Clinic (OC)**	Has a specific flow been decided to allow distancing protocol from the parking to the final appointment?			
	Does your waiting room have enough space for social distancing protocols (e.g. 4m2/patient)?			
**Discharge**	Is your regular discharge facility or rehabilitation specialist (kinesiologist, nurse…) ready to restart and receive operated patients?			
	Has a specific workflow been prepared for patient undergoing elective surgery for their postoperative regiment (see patient protection checklist)?			

**Specific concerns**	Has the facility postponed or cancelled contracts such as linen service, biohazard, waste management, outside cleaning company, shredding services, etc.?			
	Has the facility maintained daily temperature and humidity tests in ORs and sterile supply storage?			
	Has the facility been terminal cleaned?			
	Do the air exchanges need to be re-established? If yes, then follow this with terminal cleaning.			
	Have the autoclaves and other sensitive equipment been tested to verify appropriate parameters are met?			
	Are fire extinguishers still up to date?			
	Has the facility monitored daily temperatures in medication and/or tissue refrigerator?			
	Are there medications that have expired and need to be replaced?			
	Are HVAC filters compliant?			
	Has the facility kept up weekly and monthly life safety measures?			
	Has the facility taken inventory of equipment to ensure it is working properly?			
