# Supplementary material for: COVID-19 - ESSKA guidelines and recommendations for resuming elective surgery
Source: J Exp Orthop. 2020 May 13;7:28. doi: 10.1186/s40634-020-00248-4 (PMC7220621; doi:10.1186/s40634-020-00248-4)
Supplement: Supplementary file 2 — Additional file 2. What should I inform my patient about before undergoing surgery? [file 40634_2020_248_MOESM2_ESM.pdf]

ESSKA

What should I inform my patient about before undergoing surgery?

- Despite all the preventive measures you may organize within your practice/hospital to avoid a risk of COVID-19 infection during his/her surgery and/or hospitalization, the infection risk cannot be 100% excluded.

- You should inform your patients about these risks and about all aspects of his/her medical care that may change due to the current health emergency situation. You should also make sure that the patient understands these additional risks and may check with your hospital / practice administration whether an additional consent should be documented in the medical folder.

Here is a list of the different aspects that you may want to discuss with your patient (to adapt according to your practice / hospital rules). You may use it to write a specific informed consent letter adapted to your environment:

- The patient may undergo additional preoperative screening to ensure that he/she is not infected by COVID-19
- The patient should report any symptoms that may occur at any time before or after the surgery. Hiding symptoms may have dramatic consequences both for him/her and for the department (such as infecting healthcare workers or other patients)
- If a suspicion or confirmation of COVID-19 exists after the preoperative screening, the surgery may be delayed
- Despite all preventive measures, the infection risk for COVID-19 cannot be 100% excluded during surgery /hospitalization. Despite these risks, the patient still agrees to undergo surgery
- The patient understands and agrees that his/her care will be organized according to current sanitary rules:
    - Visits to the hospital / practice may be limited
    - Phone / Visio consultations may be preferred and in-person appointments with the treating doctor may be limited
    - Hospitalization time may be reduced to its strict minimum
    - Visits from family/friends during hospitalization may be limited/forbidden
    - Rehabilitation at home may be favoured
- The patient agrees to receive teleconsultations and understands the importance of organizing before his/her surgery his return to home according to the current restrictions (can receive help at home after surgery, can be easily reached by phone / Visioconference)
- The patient understands that sanitary rules within the hospital / practice may evolve according to advances of scientific knowledge on the virus and agrees to follow these rules

Published 22.04.2020
