# Supplementary material for: COVID-19 - ESSKA guidelines and recommendations for resuming elective surgery
Source: J Exp Orthop. 2020 May 13;7:28. doi: 10.1186/s40634-020-00248-4 (PMC7220621; doi:10.1186/s40634-020-00248-4)
Supplement: Supplementary file 4 — Additional file 4. Which patients should you begin with for elective orthopaedic surgery? [file 40634_2020_248_MOESM4_ESM.pdf]

Which patients should you begin with for elective orthopaedic surgery?

Priority 1: Include patients under 60, mini invasive surgery, maximum 3 days of hospital stay, no comorbidity

Priority 2: Include all patients without comorbidities, maximum 3 days of hospital stay

Priority 3: Include patients under 60, mini-invasive surgery, with comorbidities or with hospital stay >3 days

Priority 4: Include all patients with comorbidities

Priority	1		2		3		4	
Scenario	A	B	C	D	E	F	G	H
Patient age	< 60	< 60	All ages	All ages	< 60	< 60	All ages	All ages
Surgery	Mini-invasive	Mini-invasive	Mini invasive and open surgery	Mini invasive and open surgery	Mini-invasive	Mini-invasive	Mini invasive and open surgery	Mini invasive and open surgery
Length of stay	Maximum 3 days	Maximum 3 days	Maximum 3 days	Maximum 3 days	All	All	All	All
Comorbidities	None	None	None	None	None /Existing	None / Existing	Existing	Existing
COVID-19	No risk detected / Potentially recovered	Infected / Exposed With / Without symptoms Person at risk at home	No risk detected / Potentially recovered	Infected / Exposed With / Without symptoms Person at risk at home	No risk detected / Potentially recovered	Infected / Exposed With / Without symptoms Person at risk at home	No risk detected / Potentially recovered	Infected / Exposed With / Without symptoms Person at risk at home
Scheduling	Schedule surgery according to priority	Infected: Delay until full recovery for at least 6 weeks. With symptoms: Screen again in several weeks. Exposed without current symptoms: confirm results. Person at risk at home / Delay surgery if needed	Schedule surgery according to priority	Infected: Delay until full recovery for at least 6 weeks. With symptoms: Screen again in several weeks. Exposed without current symptoms: confirm results. Person at risk at home / Delay surgery if needed	Schedule surgery according to priority	Infected: Delay until full recovery for at least 6 weeks. With symptoms: Screen again in several weeks. Exposed without current symptoms: confirm results. Person at risk at home / Delay surgery if needed	Schedule surgery according to priority	Infected: Delay until full recovery for at least 6 weeks. With symptoms: Screen again in several weeks. Exposed without current symptoms: confirm results. Person at risk at home / Delay surgery if needed

Published 22.04.2020
